# Homoharringtonine Inhibits Allergic Inflammations by Regulating NF-κB-miR-183-5p-BTG1 Axis

**DOI:** 10.3389/fphar.2020.01032

**Published:** 2020-07-07

**Authors:** Misun Kim, Hyein Jo, Yoojung Kwon, Youngmi Kim, Hyun Suk Jung, Dooil Jeoung

**Affiliations:** ^1^ Department of Biochemistry, Kangwon National University, Chunchon, South Korea; ^2^ College of Medicine, Institute of New Frontier Research, Hallym University, Chunchon, South Korea

**Keywords:** anaphylaxis, atopic dermatitis, BTG1, homoharringtonine, miR-183-5p, NF-κB

## Abstract

Homoharringtonine (HHT) is a drug for treatment of chronic myeloid leukemia. However, the role of HHT in allergic inflammations remains unknown. Mouse model of atopic dermatitis (AD) induced by 2, 4,-dinitroflurobenzene (DNFB) and anaphylaxis employing 2,4-dinitropheny-human serum albumin (DNP-HSA) were used to examine the role of HHT in allergic inflammations. HHT inhibited *in vitro* allergic reactions and attenuated clinical symptoms associated with AD. DNFB induced features of allergic reactions in rat basophilic leukemia (RBL2H3) cells. HHT suppressed effect of AD on the expression of Th1/Th2 cytokines. HHT inhibited passive cutaneous anaphylaxis and passive systemic anaphylaxis. MiR-183-5p, increased by antigen stimulation, was downregulated by HHT in RBL2H3 cells. MiR-183-5p inhibitor suppressed anaphylaxis and AD. B cell translocation gene 1 (BTG1) was shown to be a direct target of miR-183-5p. BTG1 prevented antigen from inducing molecular features of *in vitro* allergic reactions. AD increased the expression of NF-κB, and NF-κB showed binding to the promoter sequences of miR-183-5p. NF-κB and miR-183 formed positive feedback to mediate *in vitro* allergic reactions. Thus, HHT can be an anti-allergy drug. We present evidence that NF-κB-miR-183-5p-BTG1 axis can serve as target for development of anti-allergy drug.

## Introduction

Homoharringtonine (HHT) exerts apoptotic effects against acute lymphoblastic leukemia (ALL) cells and chronic myeloid leukemia (CML) cells by decreasing expression of B cell lymphoma-6 (Bcl-6) (Wang et al., 2017). It decreases the tumorigenic potential of gefitinib-resistant non-small cell lung cancer cells (Cao et al., 2015), inhibits infiltration of mast tumor cells harboring imatinib-resistant D814Y KIT proto-oncogene (Jin et al., 2010), and proliferation of triple-negative breast cancer cells by decreasing the expression levels of B cell lymphoma-2 (Bcl-2), survivin, and X-linked inhibitor of apoptosis protein (XIAP) (Yakhni et al., 2019). Bcl-XL, an anti-apoptotic protein, confers protection against cytotoxic effect of HHT in human leukemia cells (Yin et al., 2011). Multicenter, noncomparative, open-label phase 2 study shows that hematologic and non-hematologic toxicity induced by subcutaneous injection of HHT is tolerable in chronic myeloid leukemia patients (Cortes et al., 2013).

HHT in combination with hsp90 inhibitor IPI504 synergistically inhibits leukemia cells by exerting apoptotic effects (Wu et al., 2019). Abivertinib, an inhibitor of Bruton's tyrosine kinase (BTK), in combination with HHT have synergistic effect in treating acute myeloid leukemia cells (Huang et al., 2019). HHT in combination with bortezomib exert cytotoxic effects against diffuse large B cell lymphoma (DLBCL) and mantle cell lymphoma (MCL) cells by myeloid leukemia cell differentiation protein-1 (MCL-1) down-regulation, Phorbol-12-Myristate-13-Acetate-Induced Protein 1 (PMAIP1) up-regulation, and Bcl-2 homologous antagonist/killer (BAK) activation (Nguyen et al., 2018).

HHT binds to NF-κB repressing factor (NKRF) (Chen et al., 2019). Second double-strand RNA-binding motif (DSRM2) domain of NKRF is necessary for binding of HHT to NKRF (Chen et al., 2019). HHT shifts NKRF from the nucleus to the cytoplasm, strengthens the p65-NKRF interaction, and interferes with p65-p50 complex formation, thereby attenuating the transactivation activity of p65 on the *MYC* gene (Chen et al., 2019). HHT binds to myosin-9 (Zhang et al., 2016). Apoptotic effect of HHT is dependent on myosin-9 (Zhang et al., 2016). HHT decreases the expression of *KIT*, a frequently mutated and/or highly expressed gene in t (8; 21) Acute Myeloid Leukemia (AML), by decreasing the expression of c-myc (Chen et al., 2019).

Anti-cancer drug candidate, AZD7762, suppresses IgE-mediated degranulation of mast cells and passive cutaneous anaphylaxis by inhibiting functions of Lyn and Fyn (Park et al., 2018). Imatinib, an inhibitor of KIT, suppresses airway hyperresponsiveness and decreases the number of activated mast cells in patients with asthma (Cahill et al., 2017). Gefitinib, an inhibitor of Epidermal growth factor receptor (EGFR) tyrosine kinase, suppresses allergic airway inflammation by inhibiting phosphatidyl inositol-3 kinase (PI3K)/Akt signaling in mouse model of asthma (Hur et al., 2007). An anti-cancer drug candidate CYC116 suppresses mast cell-mediated allergic responses (passive cutaneous anaphylaxis, passive systemic anaphylaxis) by inhibiting Fyn Kinase in mast Cells (Park et al., 2019). Anti-cancer drug ABT-737, an inhibitor of Bcl-2, suppresses clinical symptoms of atopic dermatitis (Jeong et al., 2018). HHT activates the transforming growth factor-β (TGF-β) pathway by increasing phosphorylation of smad3 (Ser423/425 (Chen et al., 2017). TGF-β inhibits allergic inflammation (Noh et al., 2017). These reports suggest that HHT may regulate allergic inflammations.

In this study, we examined roles of HHT in allergic inflammations. HHT exerted negative effects on atopic dermatitis and anaphylaxis. MiR-183-5p was one of those miRNAs that was regulated by HHT during *in vitro* allergic reactions. MiR-183-5p mediated atopic dermatitis (AD) and anaphylaxis. NF-κB was responsible for the increased expression of miR-183-5p during allergic reactions. BTG1 served as a target of miR-183-5p and inhibited *in vitro* allergic reactions. The mechanisms of anti-allergic effects by HHT merit further investigation. We provide evidence NF-κB-miR-183-5p-BTG1 axis can be a target for the development of anti-allergy drugs.

## Materials and Methods

### Materials

DNFB was purchased from Sigma-Aldrich (St. Louis, MO, United States). Anti-mouse and anti-rabbit IgG horseradish peroxidase (HRP)-conjugated antibodies were purchased from Thermo Pierce (Rockford, IL, United States). All other antibodies used in this study were purchased from Santa Cruz Biotechnology (Dallas, TX, United States). The jetPRIME transfection reagent was purchased from Polyplus (NY, United States). Oligonucleotides and primers used in this study were commercially synthesized by Bioneer Company (Daejeon, South Korea).

### Animals

Female BALB/C mice that were five week old were purchased from Nara Biotech (Seoul, South Korea). Animal experiments were approved by Institutional Animal Care and Use Committee (IACUC) of Kangwon National University (KW180823-1).

### Cell Culture

Isolations of mast cells and macrophages were performed according to the standard procedures with slight modifications (Eom et al., 2014a). Cultures were maintained in 5% CO_2_ at 37°C. Human keratinocyte HaCaT cells were purchased (HDFa lot #1780051, Gibco, Grand Island, NY, United States) and expanded in Dulbecco's modified eagle medium (Gibco, Grand Island, NY, United States) containing 8% fetal bovine serum (Gibco, Mulgrave Victoria, Australia) at 37°C with 5% CO_2_.

### MiRNA Array Analysis

The microRNA (miRNA) expression analysis was performed by using miRNA Array III kit (Signosis, CA, United States) following the manufacturer's instructions.

### MiRNA Target Analysis

TargetScan program identified targets of miR-183-5p.

### Quantitative Real-Time PCR

Total miRNA was isolated with the miRNeasy Micro Kit (Qiagen, CA, United States). The extracted miRNA was reverse transcribed using a miScript II RT Kit (Qiagen, CA, United States). The expression level of miR-183-5p was quantified with SYBR Green Master Mix (Qiagen, CA, United States) Relative expression of miRNA was calculated using the 2^-ΔΔCT^ method (ΔCT = CT_miR_ − CT_reference_).

### Transfection

For miR-183-5p knockdown, cells were transfected with 10 nM oligonucleotide (inhibitor) with Lipofectamine 2000 (Invitrogen) The sequences used were 5′-AGUGAAUUCUACCAGUGCCAUA-3′ (miR-183-5p inhibitor) and 5′-TAACACGTCTATACGCCCA-3′ (control inhibitor).

### Luciferase Activity Assays

The pGL3 3′-UTR-BTG1 construct was made by cloning a 947-bp mouse BTG1 gene segment encompassing 3′-UTR into the XbaI site of pGL3 luciferase plasmid. The mutant pGL3 3′-UTR-BTG1 construct was made with the Quick Change site-directed mutagenesis kit (Stratagene). pFlag-BTG1construct was made by PCR amplification and cloning into Flag-tagged pcDNA3.1 vector.

### Chromatin Immunoprecipitation Assays

MiR-183-5p promoter sequences, specific primers of miR-183-5p promoter-1 sequences [5′- GGCCCAGAATCTACTGATAGTG -3′ (sense) and 5′- TAAGTCTCTCTGGAGCTGGTG -3′ (antisense)], miR-183-5p promoter-2 sequences [5′- CACCAGCTCCAGAGAGACTT -3′ (sense) and 5′- AGAGGCCCAGAAGGTAAGAC -3′ (antisense)], miR-183-5p promoter-3 sequences [5′- GTCTTACCTTCTGGGCCTCT -3′ (sense) and 5′- GACTGATTTCTTGGGTTTGCAG -3′ (antisense)], and miR-183-5p promoter-4 sequences [5′- AGCCCCGTCTTTCTCCTT -3′ (sense) and 5′- CAGACCCTACAGAGAGGTCA -3′ (antisense)] were used for detection of binding of NF-κB to the promoter sequences of miR-183-5p.

### Histological Examination

Skin samples were fixed with 10% neutral buffered formalin, and embedded in paraffin. Sections (5 μm thickness) were stained with hematoxylin and eosin (H&E) or toluidine blue for leukocyte infiltration or mast cell infiltration and degranulation, respectively.

### Immunohistochemical Staining

Immunohistochemical staining of tissues was performed using an established avidin-biotin detection method (Vectastain ABC kit, Vector Laboratories Inc., Burlingame, CA, United States). The following primary antibodies were used: anti-HDAC3 (1:100, Santa Cruz Biotechnology); anti-MCP1 (1:100, Invitrogen); anti-CD163 (1: 700, Abcam); anti-TSLP (1:500, Abcam); anti-BTG1 (1:200, Abcam), and anti-inducible nitric oxide synthase (iNOS) (1: 500, Santa Cruz Biotechnology). After washing, biotinylated secondary antibody was applied at 1:100 or 1:200 dilutions for 1 h. Color was developed with diaminobenzidine (Vector Laboratories, Inc.). Sections were counterstained with Mayer's hematoxylin. The sections incubated without primary antibody served as controls. To visualize tissue mast cells, the sections were stained with 0.1% toluidine blue (Sigma) in 0.1 N HCl for 15 min.

### Immunofluorescence Staining

Cells were fixed with 4% paraformaldehyde (v/v) for 10 min and then permeabilized with 0.4% Triton X-100 for 10 min. Cells were incubated with primary antibody specific to CD163 (1:100; Abcam), iNOS (1:100; Santa Cruz Biotechnology), or NF-κB (1:200, Cell signaling Technology) for 2 h. Anti-rabbit Alexa Fluor 488 (for detection of iNOS) or anti-goat Alexa Fluor 546 (for detection of CD163 and NF-κB) secondary antibody (Molecular Probes) was added to cells and incubated for 1 h. Fluorescence images were acquired using a confocal laser scanning microscope and software (Fluoview version 2.0) with a X 60 objective (Olympus FV300, Tokyo, Japan).

### Induction of Atopic Dermatitis in BALB/C Mice

Symptoms of atopic dermatitis (AD) were induced by using DNFB, as previously described (Kwon et al., 2015). Twenty-four hours after shaving, 150 μl of 1% DNFB in acetone: olive oil mixture (3:1 vol/vol) was topically applied on days 1 through 8. Later, the same dose of 0.2% DNFB was applied four times a week. Scores of 0 (none), 1 (mild), 2 (moderate), and 3 (severe) were given for each of the four symptoms: dryness, excoriation, erosion, and erythema and edema. Clinical severity represents the sum of the individual score.

### Passive Cutaneous Anaphylaxis (PCA)

The BALB/C mice were given an intradermal injection of 2, 4-dinitrophenol (DNP)-specific IgE (0.5 μg/kg). After 24 h, the mice were intravenously injected with DNP-human serum albumin (HSA) (250 μg/kg) and 2% (v/v) Evans blue solution. After 30 min of DNP-HSA challenge, the mice were euthanized, and the 2% (v/v) Evans blue dye was extracted from each dissected ear in 700 μl of acetone/water (7:3) overnight. The absorbance of Evans blue was measured at 620 nm. To determine the effect of miR-183-5p on the passive cutaneous anaphylaxis (PCA), BALB/C mice were given an intradermal injection of IgE and intravenous injection of the indicated inhibitor (each at 100 nM). The next day, BALB/C mice were given an intravenous injection of PBS or DNP-HSA along with Evans blue solution for determining the extent of vascular permeability accompanied by PCA.

### Passive Systemic Anaphylaxis (PSA)

To determine effect of miR-183-5p on passive systemic anaphylaxis, BALB/C mice were intravenously injected with control inhibitor (100 nM) or miR-183-5p inhibitor (100 nM). The next day, BALB/C mice were given an intravenous injection of IgE (0.5 μg/kg). The next day, BALB/C mice were intravenously injected with DNP-HSA (250 μg/kg). Changes in rectal temperatures were monitored by using a digital thermometer.

### Statistical Analysis

Data were analyzed and graphed using the GraphPad Prism statistics program (Version 7, GraphPad Prism software). Results are presented as means ± SE. One–way ANOVA was carried out when multiple comparisons were evaluated. Values were considered to be significant at p value less than 0.05.

## Results

### HHT Inhibits In Vitro Allergic Inflammation

**Figure 1:**
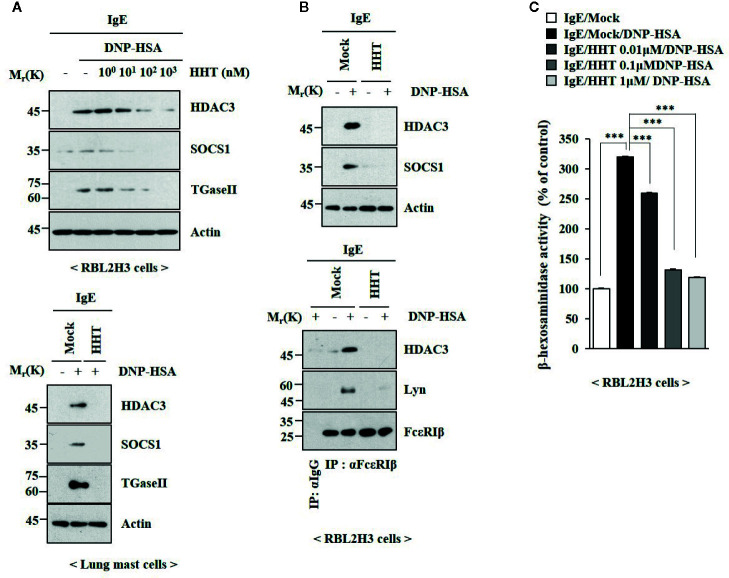
HHT inhibits *in vitro* allergic reactions. **(A)** The IgE (DNP-specific)-sensitized RBL2H3 cells (upper) or lung mast cells (lower) were treated without or with various concentrations of HHT for 1 h, followed by treatment with DNP-HSA (100 ng/ml) for 1 h. Immunoblot was performed (upper). **(B)** The IgE-sensitized RBL2H3 cells were treated without or with HHT (1 μM) for 1 h, followed by DNP-HSA stimulation for 1 h. Immunoblot and immunoprecipitation were performed (right). Immunoprecipitation employing isotype-matched anti-IgG antibody was also performed. **(C)** The IgE-sensitized RBL2H3 cells were treated without or with HHT at the indicated concentration for 1 h, followed by DNP-HSA stimulation for 1 h. ***p < 0.001.

To identify the chemical compounds that inhibit allergic inflammations, we screened chemical library, consisting of 1019 chemicals derived from natural products. We identified several compounds that prevented antigen (DNP-HSA) from increasing β-hexosaminidase activity in rat basophilic leukemia cells (RBL2H3). Among these compounds, homoharringtonine (HHT) showed the most potent effect on β-hexosaminidase activity (data not shown). HHT, in a concentration-dependent manner, prevented antigen from increasing levels Histone deacetylase 3 (HDAC3), Suppressor of cytokine signaling 1 (SOCS1) and Transglutaminase II (TGaseII) in RBL2H3 cells (**Figure 1A**). HHT showed the same effects on these molecules in lung mast cells (**Figure 1A**). Interactions of Fc epsilon receptor 1 (FcϵRI) with Src family kinase Lyn and HDAC3, induced by antigen, were inhibited by HHT (**Figure 1B**). The increased β-hexosaminidase activity, increased by antigen, was also inhibited by HHT (**Figure 1C**). The negative regulatory role of HHT in *in vitro* allergic reactions has not been reported.

### HHT Inhibits Anaphylaxis

**Figure 2:**
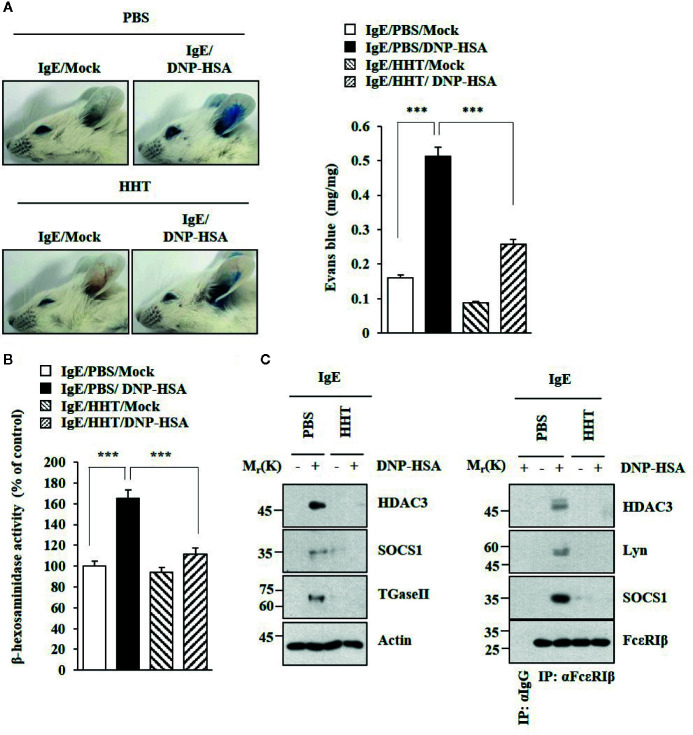
HHT inhibits PCA. **(A)** BALB/C mice were given an intradermal injection of DNP-specific IgE (0.5 μg/kg). The next day, BALB/C mice were given an intravenous injection of HHT (28 μg/kg) for 1 h, followed by an intravenous injection of PBS or DNP-HSA (250 μg/kg) along with 2% (v/v) Evans blue solution for determining the extent of vascular permeability accompanied by PCA. Representative images of each BALB/C mouse of each experimental group are shown. Each experimental group comprises four BALB/C mice. Means ± S.E. of three independent experiments are depicted. ***p < 0.001. **(B)** Ear tissue lysates were subjected to β-hexosaminidase activity assays. ***p < 0.001. **(C)** Immunoblot and immunoprecipitation were performed.

HHT exerted a negative effect on passive cutaneous anaphylaxis (PCA) employing BALB/C mouse (**Figure 2A**). HHT prevented antigen (DNA-HSA) from increasing β-hexosaminidase activity (**Figure 2B**). HHT exerted negative effects of DNP-HSA on hallmarks of allergic inflammation and interactions of FcϵRI with Lyn, HDAC3, and SOCS1 in a mouse model of PCA (**Figure 2C**). HHT prevented DNP-HSA from decreasing rectal temperature in BALB/C mouse model of passive systemic anaphylaxis (PSA) (**Figure S1A**). HHT prevented antigen from increasing β-hexosaminidase activity (**Figure S1B**) and levels of hallmarks of allergic inflammations (**Figure S1C**), and from inducing interactions of FcϵRI with Lyn and SOCS1 (**Figure S1C**). Immunohistochemical staining showed that HHT prevented antigen from increasing expression of HDAC3 (**Figure S1D**).

### HHT Inhibits AD

**Figure 3:**
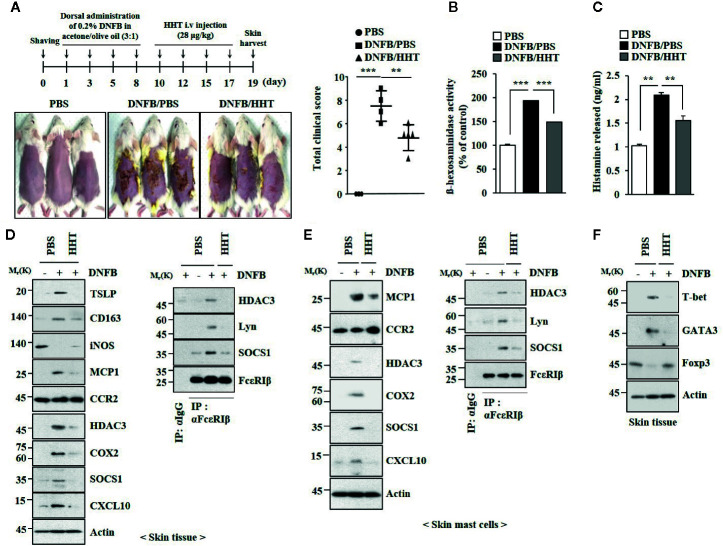
HHT attenuates clinical symptoms associated with AD. **(A)** 2, 4-dinitro fluorobenzene (DNFB) induced AD. HHT (28 μg/kg) was intravenously injected at the indicated days of the time line. Clinical scores were determined as described. Each experimental group comprises five BALB/C mice. **p < 0.01; ***p < 0.001. **(B)** β-hexosaminidase activity assays were performed. ***p < 0.001. **(C)** The amount of histamine released was determined by using sera of BALB/C mice of each experimental group. **p < 0.01. **(D)** Same as **(B)** except that immunoblot and immunoprecipitation were performed. **(E)** Same as **(D)** except that lysates of skin mast cells isolated from skin tissue were employed. **(F)** Skin tissue lysates were subjected to qRT-PCR analysis.

AD shares common molecular features with anaphylaxis (Kim et al., 2018). We examined the effect of HHT on AD. HHT suppressed clinical symptoms associated with AD induced by 2, 4,-dinitrofluorobenzene (DNFB) (**Figure 3A**). HHT exerted negative effects on the increased β-hexosaminidase activity (**Figure 3B**) and the amount of histamine released by DNFB (**Figure 3C**). HHT suppressed the effects of AD on hallmarks of allergic inflammations, such as thymic stromal lymphopoietin protein (TSLP), Cyclooxygenase 2 (COX2), Monocyte Chemoattractant Protein-1 (MCP1), and CXC chemokine ligand 10 (CXCL10) (**Figure 3D**). TSLP is a marker of AD. AD is accompanied by the increased expression of Th1 cytokines such as CXCL10 (Brunner et al., 2017). HHT suppressed the effects of AD on the expression levels of CD163 and iNOS, markers of M1 (classical) and M2 macrophages (alternative), respectively (**Figure 3D**), and the interactions of FcϵRI with Lyn, HDAC3, and SOCS1 (**Figure 3D**). HHT exerted negative effects on molecular features associated with AD (**Figure 3E**). AD increased levels of T-bet and GATA, but decreased level of Forkhead Box p3 (FoxP3) (**Figure 3F**). HHT prevented AD from increasing expression levels of HDAC3 and MCP1 (**Figure S2A**), regulating expression levels of inducible Nitric oxide synthase (iNOS) and CD163 (**Figure S2A**), inducing epidermal hyperplasia (**Figure S2B**) and increasing number of activated mast cells (**Figure S2B**). We examined whether DNFB, just like DNP-HSA, would induce features of allergic inflammation. DNFB induced features of *in vitro* allergic reactions (**Figure S3**). HHT suppressed the effects of DNFB on features of *in vitro* allergic reactions (**Figure S3**). Thus, HHT suppresses clinical symptoms of AD by regulating molecular features of allergic inflammation.

### HHT Prevents AD From Regulating Expression of Cytokines

Increased expression levels of T_H_1 (IFN-γ), T_H_2 (IL-31), and T_H_17/T_H_22 (IL-23p19/IL-8/S100A12) mRNA expression are molecular features of human AD (Guttman-Yassky et al., 2019). AD increased expression levels of Th1 cytokines such as Interleuikin-1β (IL-1β), Interferon-γ (IFN-γ) and Tumor necrosis factor- α (TNF-α) (**Figure S4**). Expression levels of Th2 cytokines, such as IL-4, IL-5, IL-6, and IL-13, were also increased by AD (**Figure S4**). AD decreased expression levels of FoxP3 and IL-10 (**Figure S4**). HHT suppressed the effects of AD on the expression levels of these cytokines. Thus, HHT suppresses AD by regulating expression levels of inflammatory cytokines.

### MiR-183-5p Mediates In Vitro Allergic Inflammation

**Figure 4:**
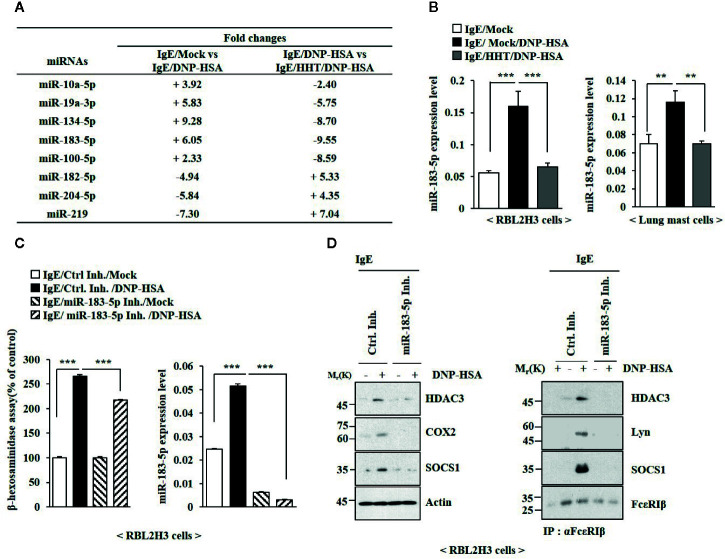
MiR-183-5p mediates allergic inflammation *in vitro*. **(A)** The IgE-sensitized RBL2H3 cells were treated without or with HHT (1 μM) for 1 h, followed by DNP-HSA stimulation for 1 h. MiRNA array analysis was performed. (+) represents increase in expression level of the indicated miRNA in RBL2H3 cells. (-) represents decrease in expression level of the indicated miRNA in RBL2H3 cells. **(B)** The IgE-sensitized RBL2H3 cells or lung mast cells were treated without or with HHT (1 μM) for 1 h, followed by DNP-HSA stimulation for 1 h. QRT-PCR analysis was performed. **p < 0.01; ***p < 0.001. **(C)** Twenty-four hours after transfection with the indicated inhibitor, cells were sensitized with IgE for 24 h, followed by DNP-HSA stimulation for 1 h. ***p < 0.001. Each inhibitor was dissolved in DMSO and diluted with PBS. **(D)** Same as **(C)** except that immunoblot and immunoprecipitation were performed.

The roles of miRNAs in allergic inflammations have been reported (Nimpong et al., 2017; Kim et al., 2019). MiRNA array analysis was employed to identify miRNAs that were regulated by HHT in RBL2H3 cells (**Figure 4A**). Among these, miR-183-5p was significantly upregulated by antigen and decreased by HHT in RBL2H3 cells (**Figure 4A**). MiR-183-5p is closely associated with occupational asthma (OA) (Lin et al., 2019). HHT exerted negative effect on the increased expression of miR-183-5p by antigen (**Figure 4B**). MiR-183-5p inhibitor suppressed the effects of antigen on the β-hexosaminidase activity (**Figure 4C**) and hallmarks of allergic inflammation (**Figure 4D**). MiR-183-5p inhibitor suppressed antigen-induced interactions involving FcϵRI (**Figure 4D**). Thus, miR-183-5p is necessary for *in vitro* allergic reactions.

### MiR-183-5p Mediates Anaphylaxis

**Figure 5:**
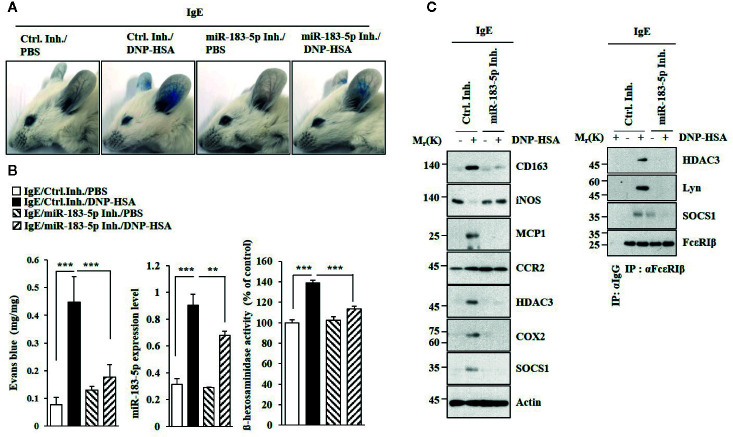
MiR-183-5p mediates PCA. **(A)** PCA was induced in the presence of the indicated inhibitor. Each experimental group comprises four BALB/C mice. **(B)** qRT-PCR and β-hexosaminidase activity assays were performed. **p < 0.01; ***p < 0.001. **(C)** Immunoblot and immunoprecipitation were performed.

MiR-183-5p inhibitor suppressed antigen-induced PCA (**Figure 5A**). PCA increased the expression of miR-183-5p (**Figure 5B**). MiR-183-5p inhibitor suppressed the effects of PCA on β-hexosaminidase activity and the amount of histamine released (**Figure 5B**). MiR-183-5p suppressed the effects of PCA on the expression levels of CD163 and iNOS, and interactions involving FcϵRI (**Figure 5C**). MiR-183-5p inhibitor suppressed the effects of antigen on rectal temperatures (**Figure S5A**) and β-hexosaminidase activity (**Figure S5B**) in BALB/C mouse model of PSA (**Figure S5A**). PSA increased the expression of miR-183-5p (**Figure S5B**). MiR-183-5p inhibitor suppressed the effects of PSA on the hallmarks of allergic inflammation, CD163, and iNOS (**Figure S5C**). MiR-183-5p inhibitor negatively regulated effect of PSA on interactions involving FcϵRI (**Figure S5C**). Thus, miR-183-5p mediates anaphylaxis.

### MiR-183-5p Mediates AD

**Figure 6:**
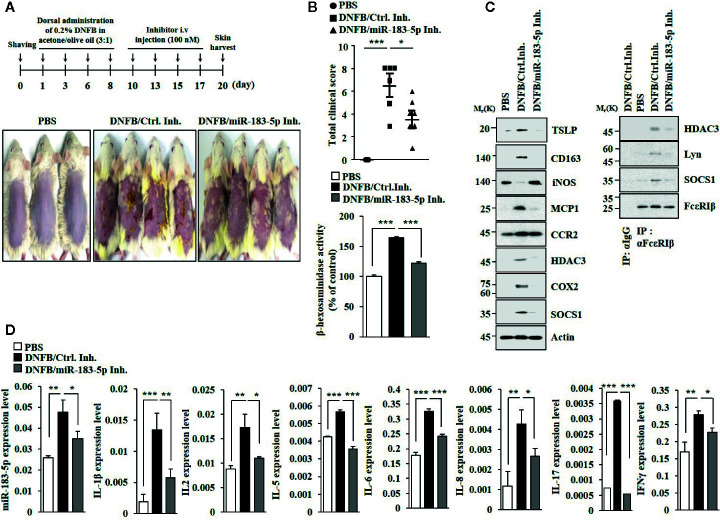
MiR-183-5p mediates AD. **(A)** AD was induced. The indicated inhibitor (each at 100 nM) was intravenously injected at the indicated days. Each experimental group comprises four BALB/C mice. **(B)** Clinical scores were determined as described (upper panel). β-hexosaminidase activity assays were performed (lower panel). *p < 0.05; ***p < 0.001. **(C)** Immunoblot and immunoprecipitation were performed. **(D)** QRT-PCR analyses were performed. *p < 0.05; **p < 0.01; ***p < 0.001.

MiR-183-5p inhibitor attenuated clinical symptoms associated with of AD (**Figure 6A**). MiR-183-5p inhibitor suppressed the effects of DNFB on β-hexosaminidase activity (**Figure 6B**), the hallmarks of allergic inflammation and CD163, and iNOS (**Figure 6C**). MiR-183-5p inhibitor suppressed the effect of DNFB on inducing interactions of FcϵRI with HDAC3, Lyn, and SOCS1 (**Figure 6C**). AD increased expression levels of IL-1β, IL-2, IL-5, IL-6, IL-8, IL-17, and IFN-γ in BALB/C mouse in a miR-183-5p-dependent manner (**Figure 6D**). MiR-183-5p inhibitor prevented DNFB from increasing expression levels of HDAC3, MCP1, and CD163, and decreasing expression of iNOS (**Figure S6A**). Toluidine blue staining showed that DNFB increased the number of activated mast cells in a miR-183-5p-dependent manner (**Figure S6B**). H&E staining showed that miR-183-5p inhibitor suppressed the effect of DNFB on skin hyperplasia (**Figure S6B**). Thus, miR-183-5p mediates AD by regulating molecular and cellular features of AD.

### NF-κB Regulates Expression of miR-183-5p

HHT binds to NF-κB repressing factor (Chen et al., 2019). AD activates NF-κB signaling in Nc/Nga mouse model (Sung and Kim, 2018). PCA activates NF-κB signaling (Kang et al., 2019). Mitogen activated protein kinase (MAPK), PI3K/AKT, and NF-κB function as downstream signaling pathways of FcϵRI (Ye et al., 2017). We hypothesized that NF-κB would be involved in the expression regulation of miR-183-5p. HHT and miR-183-5p inhibitor suppressed the effect of DNFB on the expression of NF-κB in BALB/C mouse model of AD (**Figure S7A**). HHT also prevented DNFB from increasing expression of NF-κB in RBL2H3 cells (**Figure S7A**). Toll-like receptor 2 (TLR2) activates mast cells and mediates AD (Tsurusaki et al., 2016). NF-κB signaling mediates TLR2 ligand–mediated skin inflammation (Jiao et al., 2016). DNFB increased the expression of TLR2 in BALB/C mouse and RBL2H3 cells (**Figure S7A**). HHT and miR-183-5p inhibitor suppressed the effect of DNFB on the expression of TLR2 in BALB/C mouse and RBL2H3 cells (**Figure S7A**). BAY11-7082, an inhibitor of NF-κB, prevented DNFB from inducing nuclear translocation of NF-κB in RBL2H3 cells (**Figure S7B**). NF-kB was shown to bind to the promoter sequences of miR-183-5p (**Figure S7C**). Thus, NF-κB and miR-183-5p form a positive feedback loop and mediate AD.

### BTG1 Acts as Negative Regulator of Allergic Inflammation

**Figure 7:**
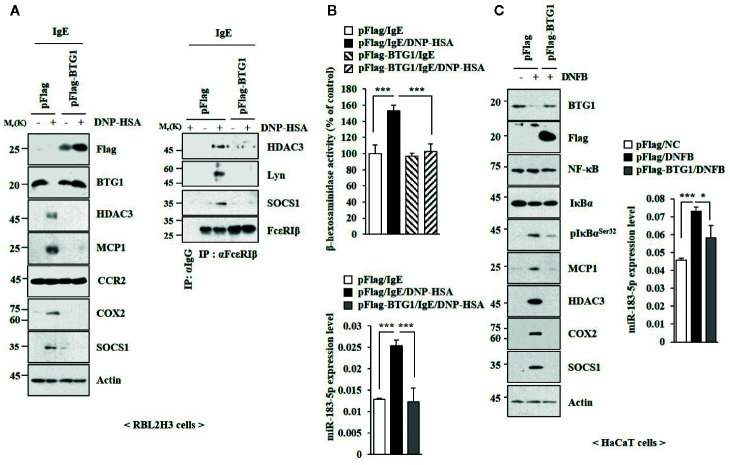
BTG1 inhibits allergic inflammation *in vitro*. **(A)** The indicated construct was transfected. The next day, cells were treated with DNP-specific IgE for 24 h, followed by treatment with DNP-HSA for 1 h. **(B)** β-hexosaminidase activity assays and qRT-PCR analysis were performed. ***p < 0.001. **(C)** Twenty-four hours after transfection, cells were treated without or with DNFB for 1 h, followed by immunoblot and qRT-PCR analysis. *p < 0.05; ***p < 0.001.

BTG1 was predicted to be a target of miR-183-5p based on TargetScan analysis. Luciferase activity assays showed the direct expression regulation of BTG1 by miR-183-5p (**Figure S8A**). HHT and miR-183-5p inhibitor suppressed the effect of DNFB on the expression of BTG1 in BALB/C mouse model of AD (**Figure S8B**). HHT prevented antigen from decreasing the expression of BTG1 in RBL2H3 cells (**Figure S8C**). BTG1 suppressed the effects of antigen on hallmarks of allergic inflammation (**Figure 7A**), interactions involving FcϵRI (**Figure 7A**), and β-hexosaminidase activity and the expression of miR-183-5p (**Figure 7B**). DNFB decreased the expression of BTG1 in HaCaT cells (**Figure 7C**). BTG1 prevented suppressed the effects of DNFB on the hallmarks of allergic inflammation and miR-183-5p in HaCaT cells (**Figure 7C**). Thus, BTG1acts as a negative regulator of *in vitro* allergic reactions.

### NF-κB Mediates In Vitro and In Vivo Allergic Inflammation

**Figure 8:**
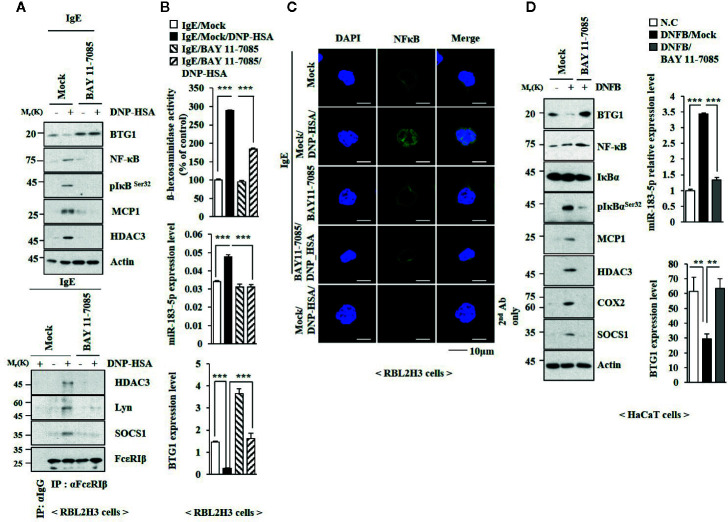
NF-κB mediates allergic reactions. **(A)** The IgE-sensitized RBL2H3 cells were pretreated without or with BAY 11-0782 (20 μM) for 1 h, followed by treatment with DNP-HSA for 1 h. BAY 11-0782 was dissolved in DMSO and diluted with PBS. **(B)** QRT-PCR analysis and β-hexosaminidase activity assays were performed. ***p < 0.001. **(C)** Immunofluorescence staining was performed. **(D)** HaCaT cells were pretreated without or with BAY 11-0782 (1 μM) for 1 h, followed by stimulation with DNFB for 1 h. Immunoblot and qRT-PCR analysis were performed. **p < 0.01; ***0p < 0.001.

TLR4-NF-κB signaling is necessary for maintaining epithelial barrier in allergic inflammation (Tao et al., 2017). BAY11-7082, an inhibitor of NF-κB, suppressed the effects of antigen on the expression levels of NF-κB, phospho I kappa B^Ser 32^ (pIkB^Ser32^), TLR2, and TLR4 (**Figure 8A**), and BTG1 in RBL2H3 cells (**Figure 8A**). BAY11-07082 also inhibited antigen-induced interactions involving FcϵRI (**Figure 8A**). BAY11-7082 suppressed the effect of antigen on the level of miR-183-5p and β-hexosaminidase activity (**Figure 8B**). BAY11-7082 rather increased the level of BTG1 mRNA in unstimulated RBL2H3 cells and prevented antigen from decreasing the level of BTG1 mRNA (**Figure 8B**). BAY11-7082 prevented antigen from inducing nuclear translocation of NF-κB (**Figure 8C**). BAY11-7082 suppressed the effect of DNFB on the expression levels of pIkB^Ser32^, hallmarks of allergic inflammation, and miR-183-5p (**Figure 8D**). BAY11-07082 prevented DNFB from decreasing the level of BTG1 in HaCaT cells (**Figure 8D**). BAY11-7082 suppressed antigen-induced PCA (**Figure S9A**) and prevented PCA from increasing β-hexosaminidase activity and the levels of BTG1 mRNA and miR-183-5p (**Figure S9B**), increasing the expression levels of BTG1, NF-κB, and pIkBα^Ser32^ (**Figure S9C**) and decreasing the expression level of BTG1 (**Figure S9C**). BAY11-7082 inhibited DNFB-induced nuclear translocation of NF-κB in HaCaT cells (**Figure S9D**).

### HHT and miR-183-5p Regulate Cellular Interactions in AD

**Figure 9:**
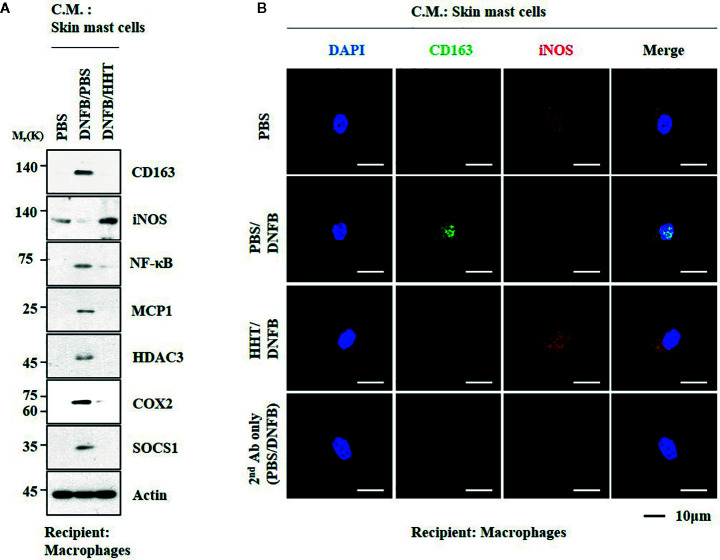
HHT regulates cellular interactions in atopic dermatitis. **(A)** The culture medium of skin mast cells isolated from BALB/C mouse was added to lung macrophages for 8 h. C.M. denotes culture medium. **(B)** Immunofluorescence staining was performed.

We examined whether cellular interactions would be necessary for AD. Culture medium of skin mast cells isolated from AD-induced BALB/C mouse increased expression levels of CD163 and hallmarks of allergic inflammation, but decreased the expression of iNOS in macrophages (**Figures 9A, B**). HHT inhibited activation of macrophages by culture medium of mast cells from AD-induced BALB/C mouse (**Figures 9A, B**). Culture medium of mast cells from AD-induced BALB/C mouse decreased expression levels of BTG1 and iNOS in macrophages (**Figure S10A, C**). Culture medium of mast cells from AD-induced BALB/C mouse injected with miR-183-5p inhibitor had no significant effect on expression level of BTG1, CD163 or iNOS in macrophages (**Figures S10A, C**). Thus, HHT and miR-183-5p inhibitor exert anti-atopic effect by inhibiting cellular interactions involving mast cells and macrophages.

## Discussion

In this study, we showed that HHT exerted a negative effect on the increased expression of HDAC3 by antigen in RBL2H3 cells. HDAC3 mediates allergic skin inflammation by increasing expression level of MCP1 and binds to FcϵRI (Kim et al., 2012). HDAC3 mediates tumorigenic and metastatic potential enhanced by passive systemic anaphylaxis (Eom et al., 2014b). HDAC3 exerts negative effect on the expression of HDAC2 in antigen-stimulated RBL2H3 cells (Kim et al., 2012).

HHT prevented AD and anaphylaxis from increasing expression of SOCS1. SOCS1, decreased by TGFβ, mediates *in vitro* and *in vivo* allergic inflammation and binds to FcϵRI (Noh et al., 2017). SOCS1 regulates AD by forming a negative feedback loop with miR-122a-5p (Kim et al., 2018). It is reasonable that HHT may increase expression level of TGFβ and miR-122a-5p.

AD is accompanied by an increased expression levels of Th2 cytokines such as IL-4, IL-5, and IL-13 (Wang et al., 2018). In mouse model of allergic lung disease, inhibition of pulmonary Th2 responses leads to increased production IL-10 (Nunes et al., 2019). TSA, an inhibitor of HDACs, suppresses DNFB-induced AD by decreasing production of Th2 cytokines such as IL-4 (Kim et al., 2010). We showed that AD increased expression levels of TH1/TH2 cytokines but decreased the expression of IL-10. HHT prevented AD from increasing expression level of IL-17. IL-17 is closely associated with asthma and AD development (Silva et al., 2019). IL-17 allows F-actin to interact with myosin and is critical for the contraction of airway smooth muscle cells (Bulek et al., 2019). Decreased expression of IL-17 leads to resolution of psoriasis (Krueger et al., 2019). IL-17 is necessary for the pathogenesis of allergic airway hyperresponsiveness (Bulek et al., 2019). It would be necessary to examine whether IL-17 would regulate AD and anaphylaxis.

We identified the miRNAs that were regulated by HHT. MiR-183-5p was one of those miRNAs that were increased by antigen stimulation and downregulated by HHT in RBL2H3 cells. MiR-183-5p targets hemeoxygenase 1(HO-1) (Davis et al., 2017). HO-1 suppresses Th2 responses in a mouse model of eosinophilic asthma (Lv et al., 2018). Nuclear receptor subfamily 2 (Nrf2)/HO-1 signaling negatively regulates degranulation in mast cells (Ye et al., 2017). In this study, we showed that miR-183-5p mediated AD and anaphylaxis.

TargetScan analysis predicted BTG-1 as a target of miR-183-5p. MiR-183-5p inhibitors acts as a negative regulator of BTG1 and suppresses endothelial cell tube formation (Zhang et al., 2019). BTG1 acts as a negative regulator of intestinal inflammation (He et al., 2017). In this study, we showed that overexpression of BTG1 inhibited *in vitro* allergic reactions. The roles of BTG1 in allergic inflammations have not been reported.

In this study, we found that miR-19a-3p was also regulated by HHT in antigen-stimulated RBL2H3 cells. MiR-19a-3p targets BTG1 and regulates apoptosis of castration-resistant prostate cancer cells (Lu et al., 2015). MiR-10a-5p was also regulated by HHT in antigen-stimulated RBL2H3 cells. MiR-10a-5p acts as a negative regulator of metastatic potential of colorectal cancer cells (Liu et al., 2017). miR-100 suppresses inflammatory activation of microglia by inactivating TLR4-NF-κB signaling pathway (Li et al., 2019). miR-134-5p attenuates neuropathic pain by directly decreasing the expression of Twist1 (Ji et al., 2018). miR-182 promotes osteoclastogenesis by directly regulating protein kinase double-stranded RNA-dependent (PKR) in mouse model of osteoporosis and inflammatory arthritis (Inoue et al., 2018). Overexpression of miR-204 induces apoptosis and decreases the expression levels of gens involved in ER stress response in human trabecular meshwork cells (Li et al., 2011). miR-219 promotes spinal cord injury recovery by inhibiting Neuro D2-mediated inflammation (Zhu et al., 2019). Roles miRNAs in allergic inflammations remain to be seen.

Autophagy is necessary for degranulation of mast cells (Nakano and Ushio, 2011). Mast cell degranulation during anaphylaxis requires autophagy related-7 (ATG-7), a marker of autophagy (Ushio et al., 2011). It would be necessary to examine whether NF-κB-miR-183-5p-BTG1 axis can regulate autophagic flux during allergic inflammation.

Anaphylaxis involves cellular interactions among mast cells, macrophages, and endothelial cells (Eom et al., 2014b; Kim et al., 2019). In this study, we showed that culture medium of activated skin mast cells increased hallmarks of allergic inflammation and CD163. This suggests that soluble factor(s) may mediate interactions between mast cells and macrophages. Exosomes mediate cellular interactions during anaphylaxis (Kim et al., 2019). Exosomes of BALB/C mouse under AD might induce molecular features of AD in BALB/C mouse. Bone marrow-derived exosomes contain miR-183-5p (Davis et al, 2017). It would be necessary to examine the presence miR-183-5p in the exosomes of BALB/C mouse under AD and also examine exosomal miRNAs that could regulate molecular features of AD in BALB/C mouse. It is probable that HHT may prevent cellular interactions mediated by exosomes during allergic inflammations.

High expression of histamine H1repceptor (H(1)R) is found in patients with allergic rhinitis (Islam et al., 2018). HHT is an activator of H(I) R (Guo et al., 2014). This suggest that HHT can act as a potential allergen. In this study, we showed that HHT itself did not induce features of passive cutaneous anaphylaxis (**Figure 2**) or passive systemic anaphylaxis (**Figure S1**). HHT did not induce molecular features of allergic inflammation in RBL2H3 cells or mast cells (**Figure 1**). In mouse model of AD, HHT did not cause clinical symptoms of AD (data not shown).

For better understanding the mechanism of anti-allergic effect of HHT, identification of proteins that bind to HHT will be necessary. For this, biotin labeling of HHT will make it possible to identify HHT-binding proteins. Incubation of cell lysates with biotin-labeled of HHT, followed by electrospray-ionization quadrupole time-off light mass spectrometry (ESI-Q-TOF MS) will make it possible to identify HHT-binding proteins (Chen et al., 2019). Binding DB (www.bindingdb.org) predicts that Egl nine homolog1 (PHD2), porphobilinogen synthase, Glycosylasparaginase (GA), Histone lysine demethylase or Methyl-accepting chemotaxis protein(McpS) can bind to HHT. It will be necessary to examine roles of these proteins in allergic inflammations.

Chemotherapy can cause skin toxicities such as Stevens-Johnson syndrome (SJS) and toxic epidermal necrolysis (TEN) (Tanaka et al., 2019). It is possible that HHT may cause some skin toxicity. Anti-cancer drugs can cause skin toxicities such as paronychia, acneiform eruption, and alopecia, SJS and toxic epidermal necrolysis TEN (Ng et al., 2018). Prolonged treatment of anti-cancer drugs may cause death of normal cells *via* non-specific mechanisms and cause side effects (Park et al., 2019). In order to suppress atopic dermatitis, it would be necessary to apply HHT as the topical mediation for a short period time rather than oral administration. HHT is converted into HHT-acid form by human plasma (Ni et al., 2003). HHT-acid form is 700-fold time less toxic than HHT (Ni et al., 2003). Application of HHT-acid form as the topical medication may suppress atopic dermatitis without toxic effects. It is also necessary to compare the effect of intravenous injection of HHT-acid to the topical application of HHT-acid.

In this study, we showed potential of HHT as an anti-allergic drug. We also presented evidence that NF-κB-miR-183-5p-BTG1 axis mediates AD and anaphylaxis. For better understanding mechanism of anti-atopic effect of HHT, it would be necessary to identify molecules that regulate the expression levels of genes that are involved in NF-κB-miR-183-5p-BTG1 axis.

## Data Availability Statement

The raw data supporting the conclusions of this article will be made available by the authors, without undue reservation, to any qualified researcher.

## Ethics Statement

The animal study was reviewed and approved by Institutional Animal Care and Use Committee (IACUC) of Kangwon National University.

## Author Contributions

DJ and HSJ conceived and designed this study. MK, HC, and YKw performed experiments and analyzed the data. DJ and HSJ drafted and revised the manuscript. YKi provided suggestion for the experiments. All authors contributed to the article and approved the submitted version.

## Funding

This work was supported by National Research Foundation Grants (2017M3A9G7072417, 2018R1D1A1B07043498, 2020R1A2C1006996), a grant from the BK21 plus Program.

## Conflict of Interest

The authors declare that the research was conducted in the absence of any commercial or financial relationships that could be construed as a potential conflict of interest.
