# Supplementary material for: Homoharringtonine Inhibits Allergic Inflammations by Regulating NF-κB-miR-183-5p-BTG1 Axis
Source: Front Pharmacol. 2020 Jul 7;11:1032. doi: 10.3389/fphar.2020.01032 (PMC7358642; doi:10.3389/fphar.2020.01032)
Supplement: Supplementary file 1 [file DataSheet_1.pdf]

Supporting Information

Homoharringtonine inhibits allergic inflammations by regulating NF-κB- miR-183-5p-BTG1 axis

Misun Kim[1], Hyein Jo[1], Yoojung Kwon[1], Youngmi Kim[2], Hyun Suk Jung[1] and Dooil Jeoung[1,*]

[1] *Department of Biochemistry, Kangwon National University, Chunchon 24341, Korea*
[2] *Institute of New Frontier Research, College of Medicine, Hallym University, Chunchon 24252, Korea*

Figure S1

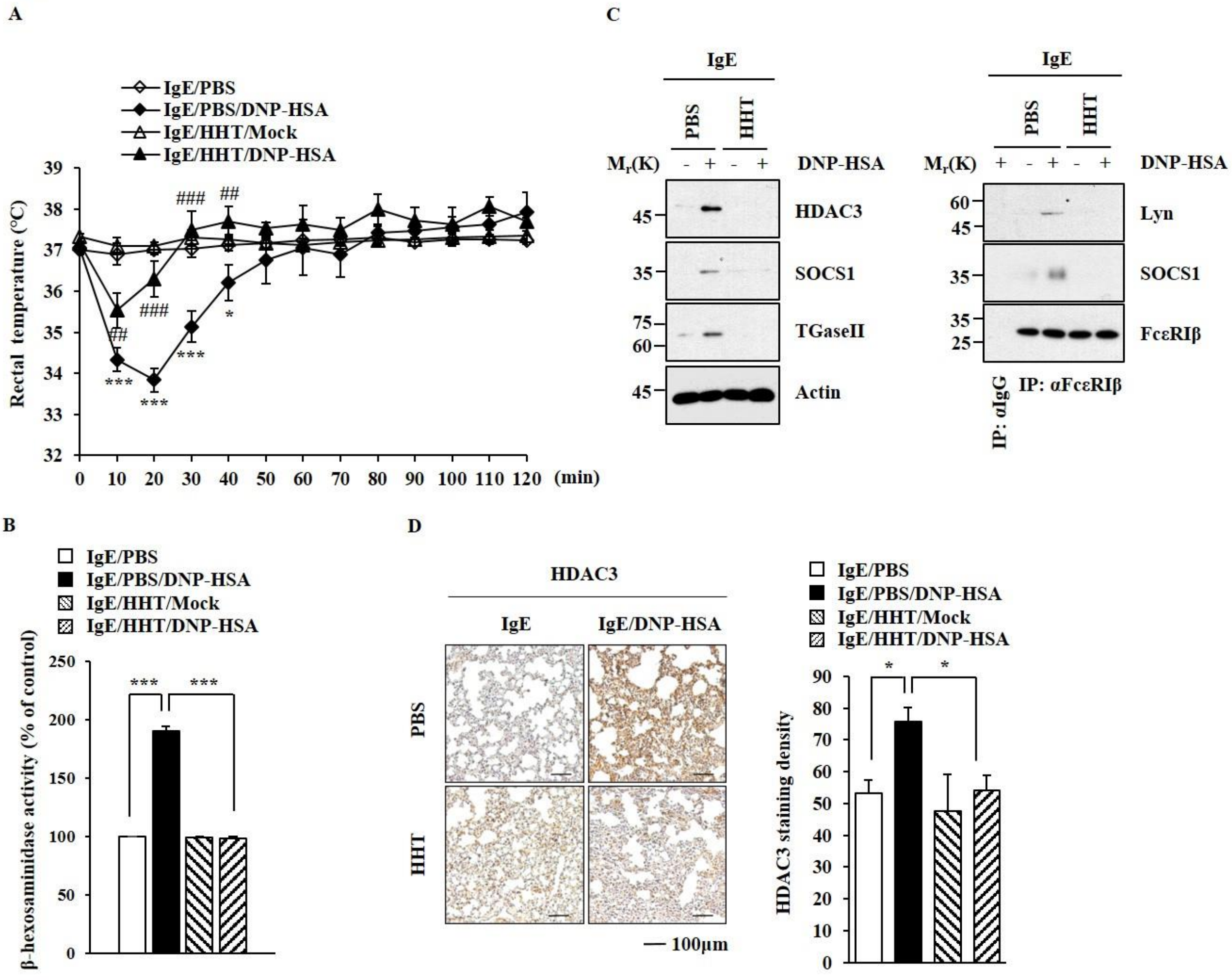

FIGURE S1. **HHT inhibits PSA.** (A) DNP-specific IgE (0.5 μg/kg) was intravenously injected into BALB/C mice. Twenty-four hours later, HHT (28 μg/kg) was intravenously injected into BALB/C mice for 1 h. One hour later, BALB/C mice were given an intravenous injection of DNP-HSA (250 μg/kg). At each time point after injection with DNP-HSA, rectal temperatures were measured. Each experimental group comprises five mice. *, $p<0.05$; ***, $p<0.001$, compared with IgE/PBS; ##, $p<0.01$; ###, $p<0.001$, compared with IgE/PBS/DNP-HSA. (B) β-hexosaminidase activity assays were performed. (C) Immunoblot and immunoprecipitation were performed. (D) Immunohistochemical staining employing lung tissue was performed. Quantification was performed by calculating the percentage of the staining intensities using Image J (NIH). *, $p<0.05$.

Figure S2

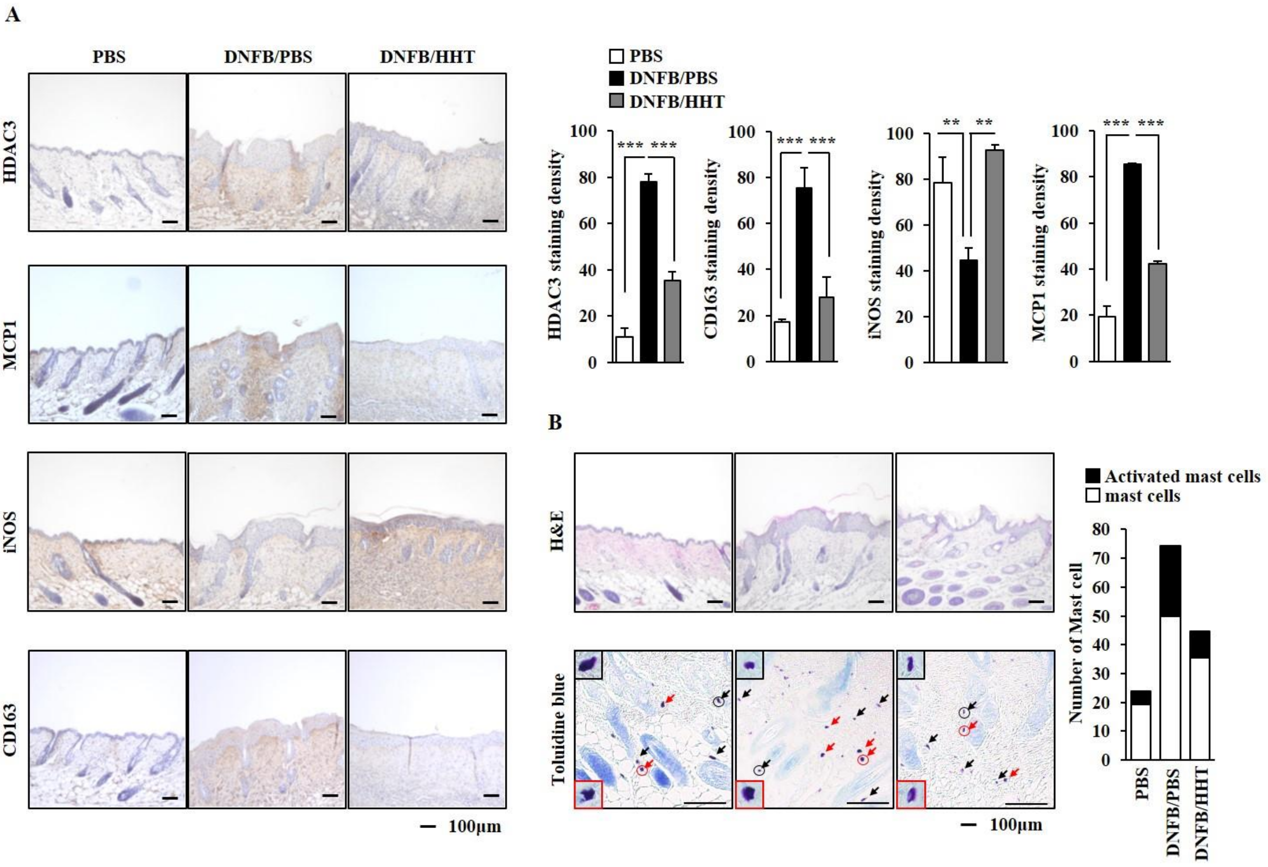

FIGURE S2. HHT inhibits molecular and cellular features of AD. (A) Immunohistochemical staining was performed as described. Quantification was performed by calculating the percentage of the staining intensities using Image J (NIH). **, p<0.01; ***, p<0.001. (B) H&E staining was performed to examine the extent of epithelial hyperplasia (upper). Toluidine blue staining was performed to examine the number of activated mast cells (lower). Red arrows denote activated mast cells. Red rectangle is an enlarged version of activated mast cells (red circle). Black rectangle is an enlarged version of mast cells (black circle).

Figure S3

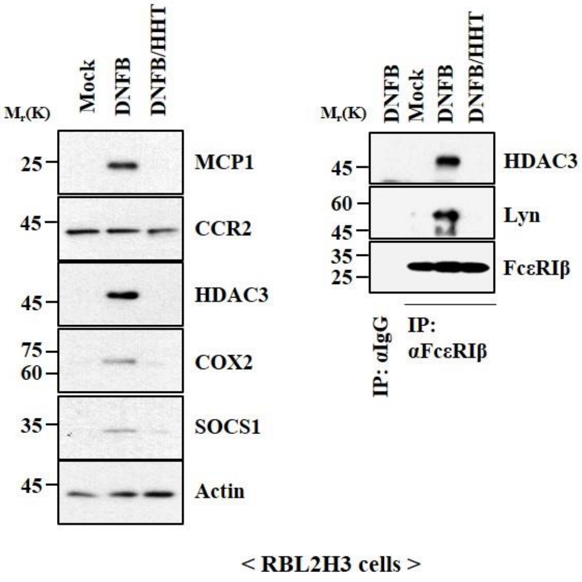

FIGURE S3. DNFB induces features of allergic inflammation. RBL2H3 cells were pretreated without or with HHT (1 μM) for 1 h, followed by DNFB (100 nM) treatment for 1 h. Immunoblot and immunoprecipitation were performed.

Figure S4

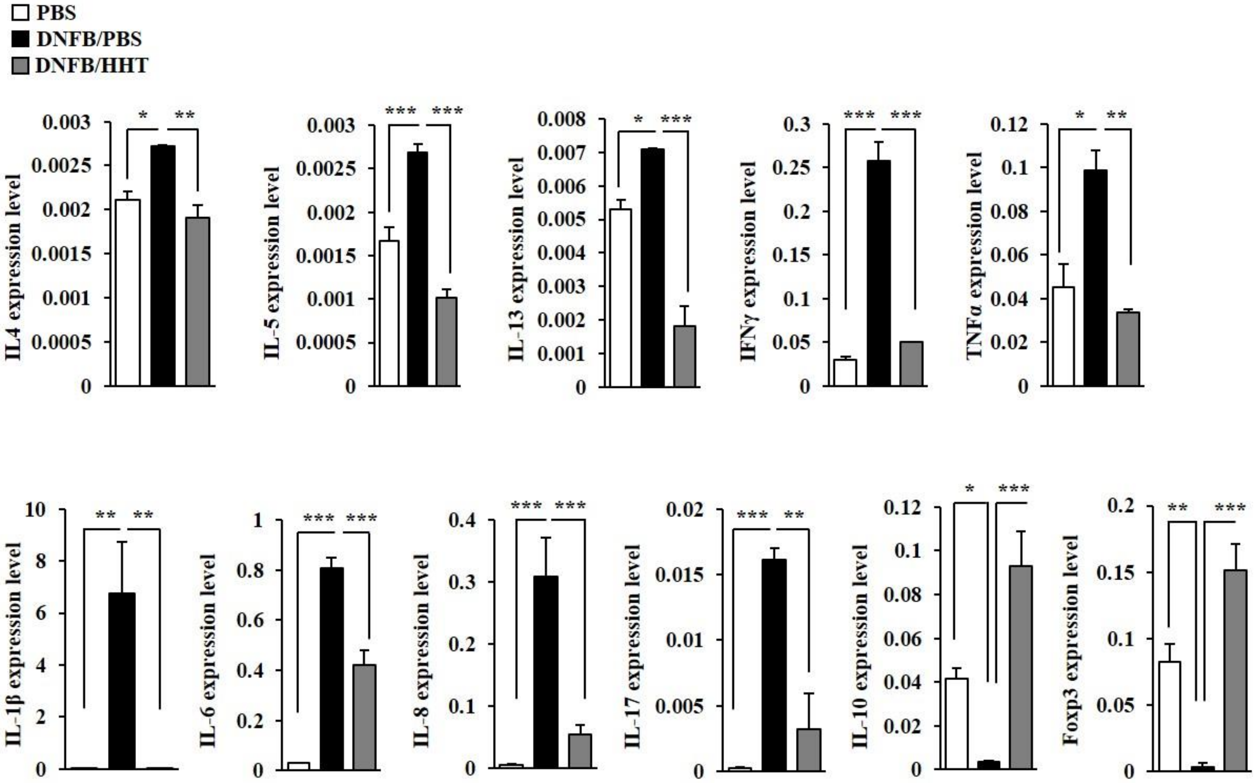

FIGURE S4. HHT prevents antigen from regulating expression levels of TH1/TH2 cytokines and FoxP3 in AD. QRT-PCR analysis was performed. *, P<0.05; **, p<0.01; ***, p<0.001.

Figure S5

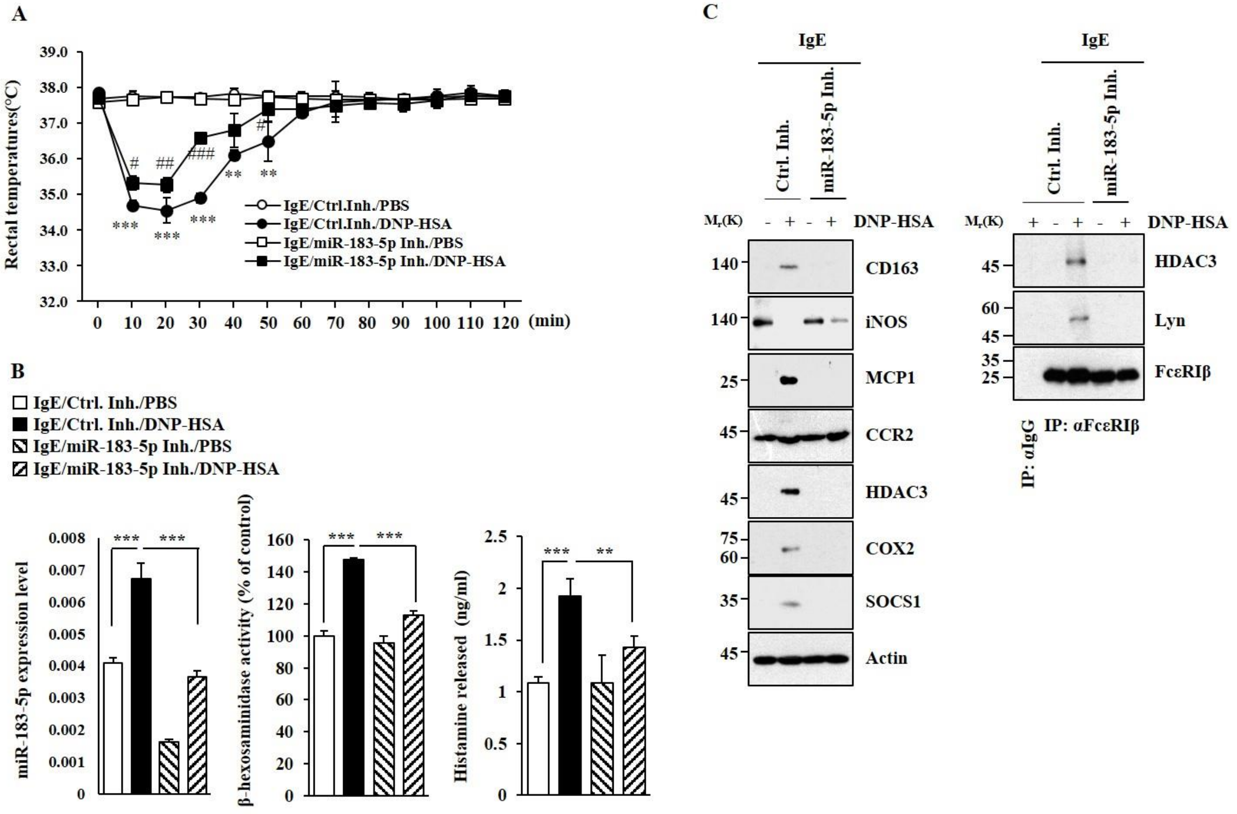

FIGURE S5. MiR-183-5p mediates PSA. (A) PSA was induced in the presence of the indicated inhibitor (each at 100 nM). The next day, PBS or DNP-HSA (250 μg/kg) was intravenously injected into BALB/C mice. Each experimental group comprises four BALB/C mice. **, p<0.01; ***, p<0.001, compared with IgE/Ctrl. Inh./PBS; #, p<0.05; ##, p<0.01; ##, p<0.001, compared with IgE/Ctrl. Inh./DNP-HSA. (B) β-hexosaminidase activity assays and qRT-PCR analysis were performed. The amount of histamine released was also determined. **p < 0.01; ***, p<0.001. (C) Immunoblot and immunoprecipitation were performed.

Figure S6

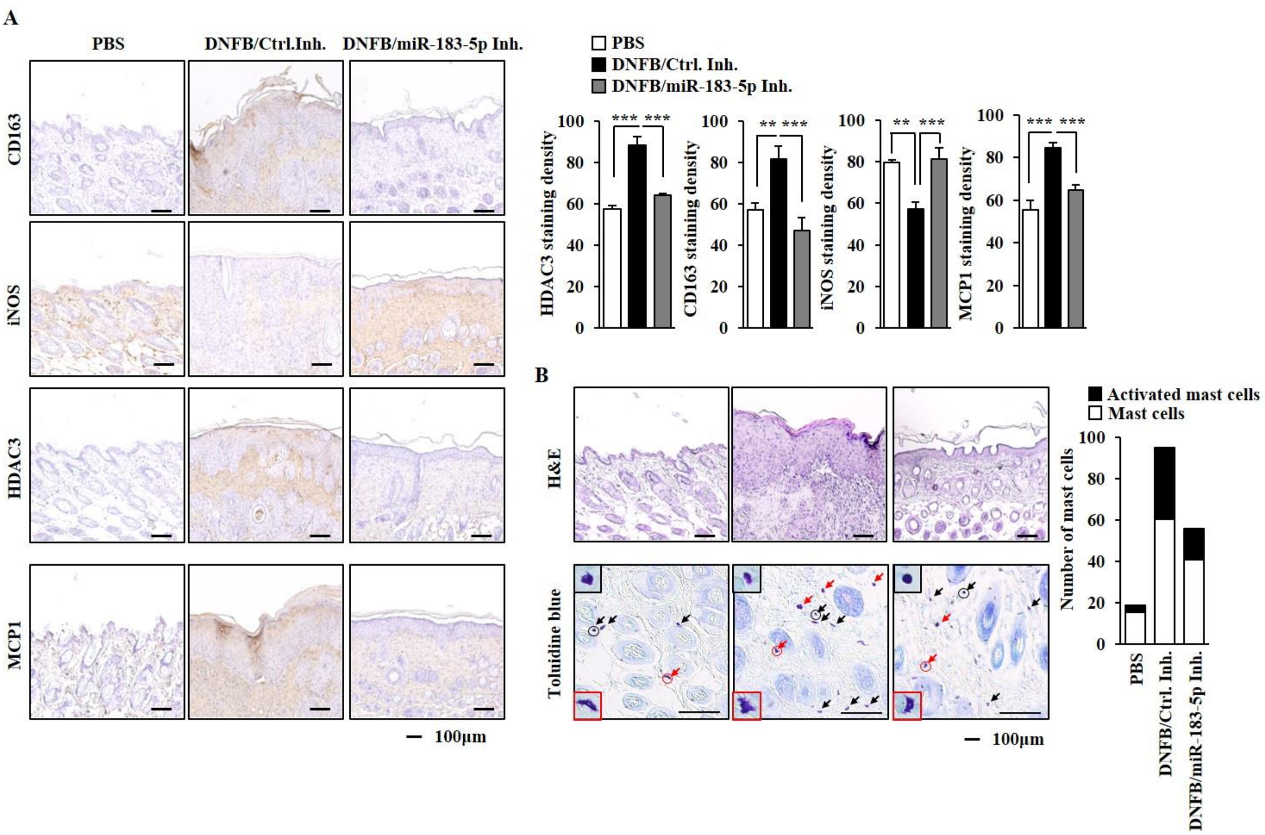

FIGURE S6. MiR-183-5p inhibitor negatively regulates features of AD. (A) Immunohistochemical staining of skin tissue of each mouse of each experimental group was performed. Quantification was performed by calculating the percentage of the staining intensities using Image J (NIH). **, $p < 0.01$; ***, $p<0.001$. (B) H&E staining (upper panel) and toluidine blue staining (lower panel) were performed. Red arrows denote activated mast cells. Red rectangle is an enlarged version of activated mast cells (red circle). Black rectangle is an enlarged version of mast cells (black circle).

Figure S7

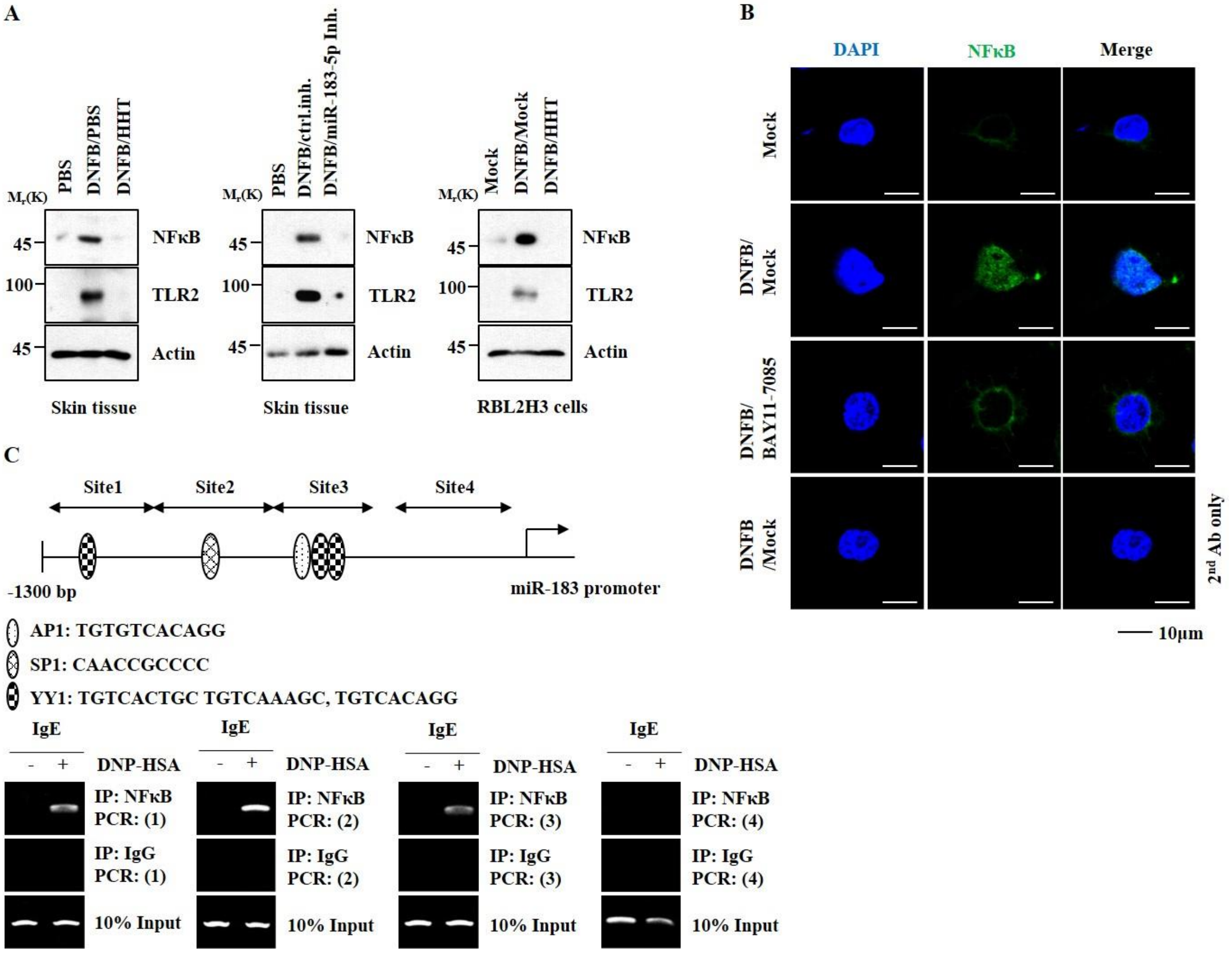

FIGURE S7. MiR-183-5p and NF-kB form positive feedback loop to mediate allergic inflammation. (A) Skin tissue lysates from BALB/C mouse under AD without or with HHT (left) or BALB/C mouse under AD with the injection of the indicated inhibitor (middle) were subjected to immunoblot (middle). RBL2H3 cells were treated without or with HHT (1 μM) for 1 h, followed by DNFB (100 μM) treatment for 1 h (right). (B) RBL2H3 cells were treated without or with HHT for 1 h, followed by DNFB treatment for 1 h. Immunofluorescence staining was performed. (C) ChIP assays were performed a described. Numbers in parentheses denote PCR-amplified regions.

Figure S8

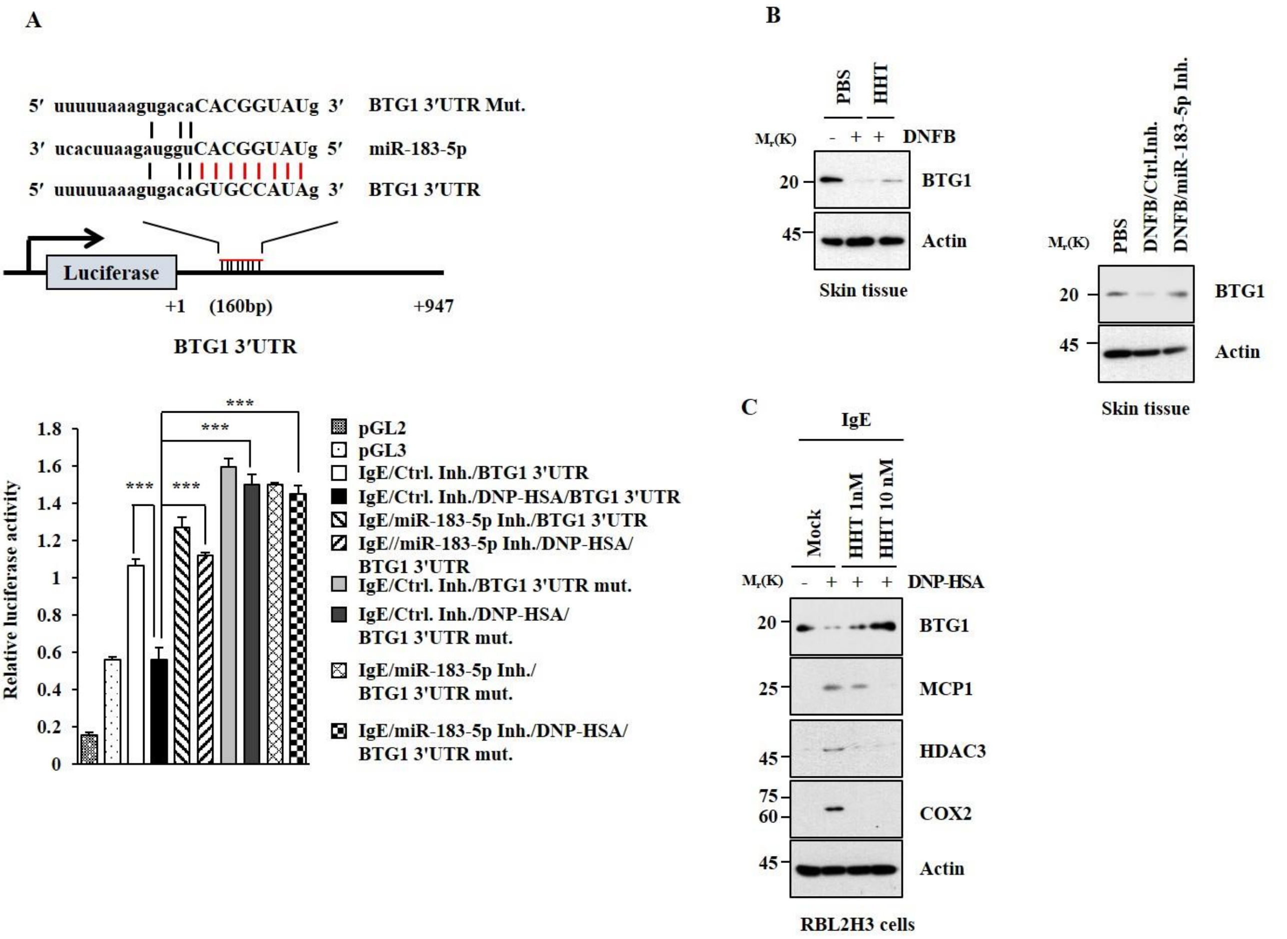

FIGURE S8. MiR-183-5p directly targets BTG1. (A) Potential binding of miR-183-5p to the 3′-UTR of BTG1 was shown. Wild type Luc-BTG1-3′-UTR or mutant Luc-BTG1-3′-UTR was transfected along with the indicated inhibitor (each at 10 nM) into the indicated cell line. Luciferase activity assays were performed as described. ***, p<0.001. (B) Skin tissue lysates from BALB/C mice under AD were subjected to immunoblot. (C) The IgE-sensitized RBL2H3 cells were pretreated without or with HHT at the indicated concentration for 1 h.

Figure S9

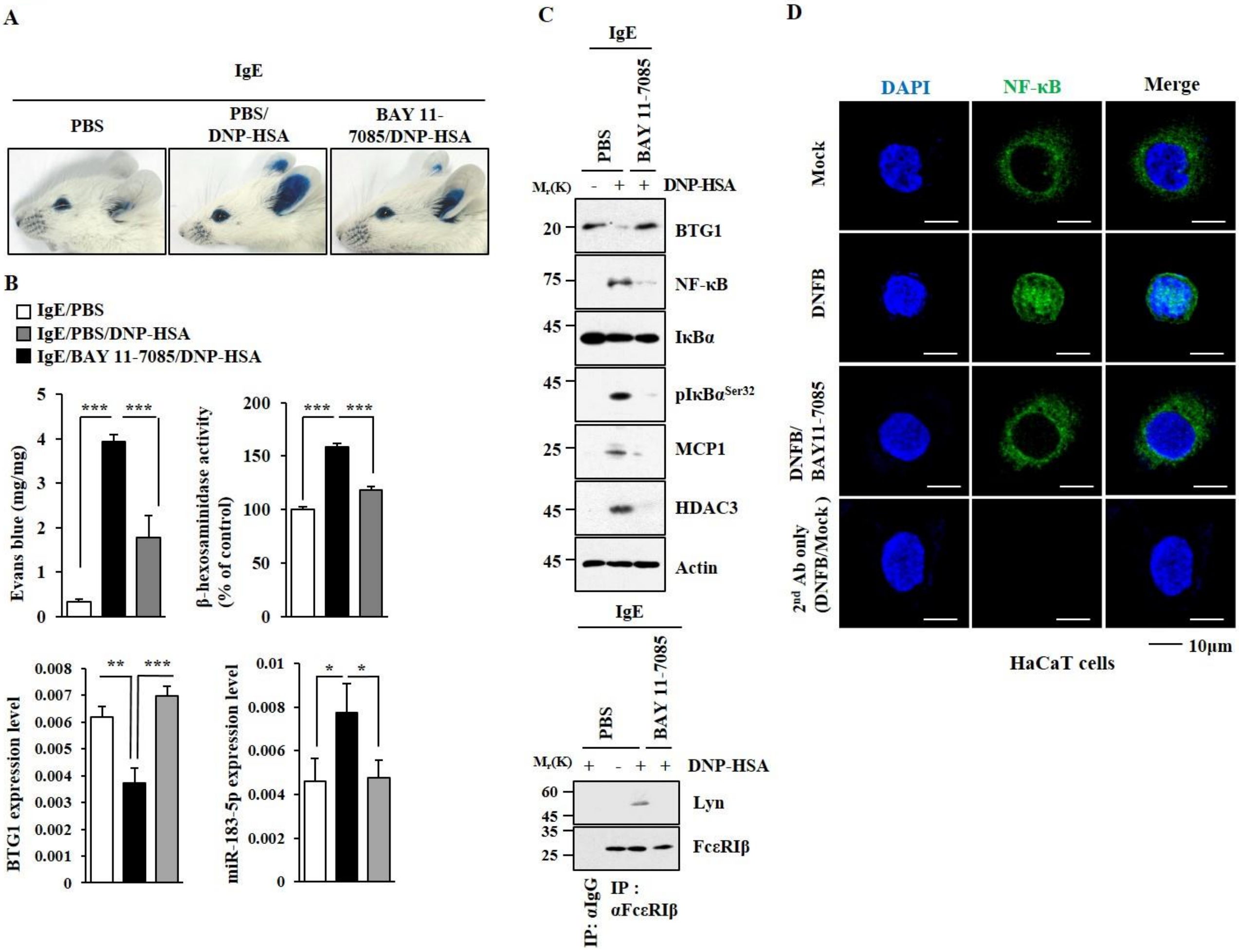

FIGURE S9. NF-kB mediates PCA. (A) Twenty-four hours after intradermal injection of DNP-specific IgE antibody (0.5 μg/kg) or IgG (0.5 μg/kg), BALB/C mice were given an intravenous injection of PBS or DNP-HSA along with BAY 11-0782 (1 mg/kg) and Evans blue solution. Each experimental group copmrises four BALB/C mice. (B) β-hexosaminidase activity assays and qRT-PCR analysis were performed. *, $p<0.05$; **$p < 0.01$; ***, $p<0.001$. (C) Immunoblot and immunoprecipitation were performed. (D) HaCaT cells were pretreated without or with BAY 11-0782 (1 μM) for 1 h, followed by stimulation with DNFB for 1 h.

Figure S10

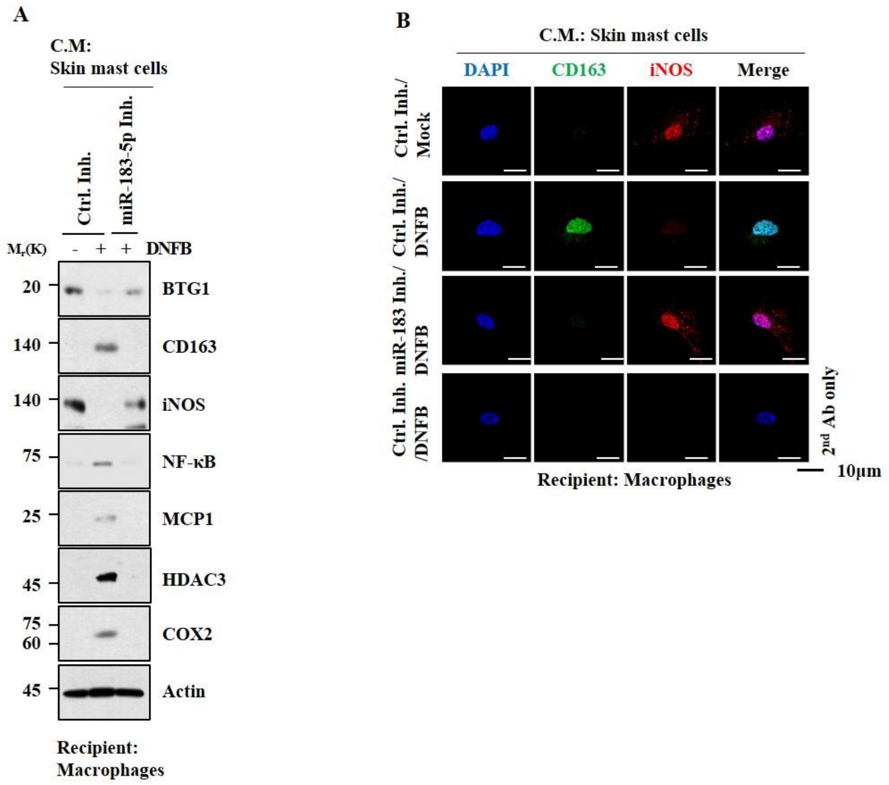

FIGURE S10. miR-183-5p inhibitor inhibit cellular interactions during AD. (A) The culture medium of skin mast cells isolated from BALB/C mouse was added to lung macrophages for 8 h. (B) Immunofluorescence staining was performed.
